# In vivo GDF3 administration abrogates aging related muscle regeneration delay following acute sterile injury

**DOI:** 10.1111/acel.12815

**Published:** 2018-07-12

**Authors:** Andreas Patsalos, Zoltan Simandi, Tristan T. Hays, Matthew Peloquin, Matine Hajian, Isabella Restrepo, Paul M. Coen, Alan J. Russell, Laszlo Nagy

**Affiliations:** ^1^ Sanford-Burnham‐Prebys Medical Discovery Institute at Lake Nona Orlando Florida; ^2^ Department of Biochemistry and Molecular Biology, Faculty of Medicine University of Debrecen Debrecen Hungary; ^3^ Florida Hospital Translational Research Institute for Metabolism and Diabetes Orlando Florida; ^4^ Muscle Metabolism Discovery Performance Unit GlaxoSmithKline King of Prussia Pennsylvania

## Abstract

Tissue regeneration is a highly coordinated process with sequential events including immune cell infiltration, clearance of damaged tissues, and immune‐supported regrowth of the tissue. Aging has a well‐documented negative impact on this process globally; however, whether changes in immune cells per se are contributing to the decline in the body’s ability to regenerate tissues with aging is not clearly understood. Here, we set out to characterize the dynamics of macrophage infiltration and their functional contribution to muscle regeneration by comparing young and aged animals upon acute sterile injury. Injured muscle of old mice showed markedly elevated number of macrophages, with a predominance for Ly6C^high^ pro‐inflammatory macrophages and a lower ratio of the Ly6C^low^ repair macrophages. Of interest, a recently identified repair macrophage‐derived cytokine, growth differentiation factor 3 (GDF3), was markedly downregulated in injured muscle of old relative to young mice. Supplementation of recombinant GDF3 in aged mice ameliorated the inefficient regenerative response. Together, these results uncover a deficiency in the quantity and quality of infiltrating macrophages during aging and suggest that in vivo administration of GDF3 could be an effective therapeutic approach.

## 1 INTRODUCTION, RESULTS, DISCUSSION

Skeletal muscle mass, function, and capacity to repair upon injury, all progressively decline with aging resulting in restrictions to mobility, voluntary function, metabolism, and eventually quality of life. In adult tissues, satellite cells are kept in a quiescent state until they are activated to regenerate damaged muscle through cycles of self‐renewal divisions (reviewed in (Montarras, L'Honoré, & Buckingham, 2013)). The ability of satellite cells to repair injured muscle markedly declines with aging (Cheung & Rando, 2013). Aside from reduced numbers of satellite cells (Garcia‐Prat, Sousa‐Victor, & Munoz‐Canoves, 2013; Shefer, Mark, Richardson, & Yablonka‐Reuveni, 2006), the differentiation capacity of satellite cells is also reduced with aging. Moreover, the number of differentiating satellite cells is decreased in aged mice, as shown by downregulation of differentiation markers such as desmin and myogenin (Charge, Brack, & Hughes, 2002; Collins, Zammit, Ruiz, Morgan, & Partridge, 2007). In addition to satellite cells, there is clear evidence supporting the essential role of immune cells both in the clearance of damaged tissue and enhancing tissue regeneration upon injury (Tidball, 2017). However, age‐related changes in the immune cell functions and its therapeutic potential remain elusive. Here, we demonstrate that innate immune cells are an important component of age‐related delay in muscle regeneration. As a proof of concept, we show that the number of reparative macrophages and the level of growth differentiation factor 3 (GDF3) produced by these cells are severely decreased with aging in regenerating muscles, leading to delayed repair. Supplementation of the cytokine alone can restore the normal recovery time following acute injury, and thus, it provides a new therapeutic approach to treat muscle injury in elderly people.

**Figure 1:**
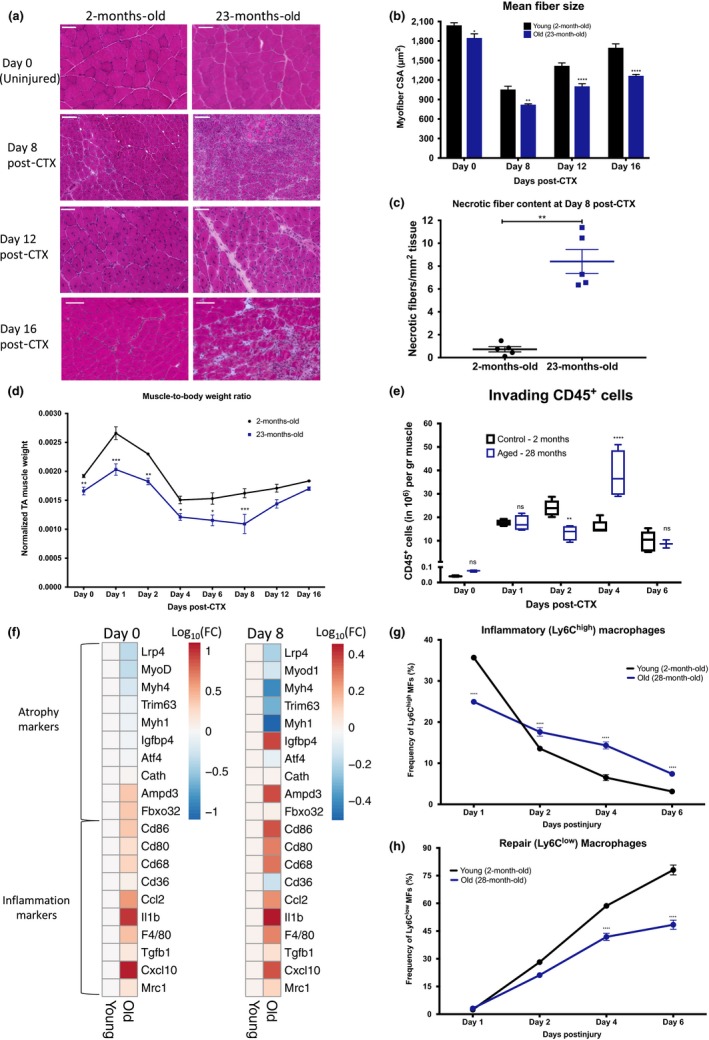
Impaired skeletal muscle regeneration and delayed phenotypic transition of infiltrating myeloid cells in aged animals following CTX injury. (a) Representative images of H&E‐stained skeletal muscle from young adult (2‐month‐old) and aged (23‐month‐old) male mice at Days 0, 8, 12, and 16 post‐CTX‐induced injury. Scale bars in the upper left represent 100 μm. (b) Mean myofiber cross‐sectional area (CSA) of regenerating muscles in young adult (2‐month‐old) and aged (23‐month‐old) mice (number of fibers counted > 20,000) at Days 0, 8, 12, and 16 post‐CTX‐induced injury (*n* = 6 per group). (c) The ratio of necrotic fibers relative to regeneration area (in mm^2^) at Day 8 of regeneration in young adult (2‐month‐old) and aged (23‐month‐old) muscle sections is shown. (d) Normalized *tibialis anterior* (TA) muscle mass‐to‐body weight ratio from young adult (2‐month‐old) and aged (23‐month‐old) mice at indicated time points following CTX injury (*n* = 6 per group). (e) Number of infiltrating myeloid (CD45^+^) cells in regenerating muscle from young (2‐month‐old) or aged (28‐month‐old) muscles at indicated time points prior and post‐CTX injury (*n* = 8 muscles per group). (f) Heatmap representations of atrophy and macrophage‐related genes (measured by qPCR) from young and old uninjured (left panel) and regenerating (Day 8 post‐CTX; right panel) TA muscles. Relative mRNA expression (calculated using the 2^‐ΔΔCT^ method) is shown as log_10_(fold change) (*n* = 6 muscles per group). (g and h) Percentage of inflammatory (Ly6C^high^ F4/80^low^) and repair (Ly6C^low^ F4/80^high^) MFs from young (2‐month‐old) or aged (28‐month‐old) muscles at indicated time points following CTX injury (*n* = 8 mice per group). In all bar and line graphs, bars and data points represent mean ± *SEM*

In line with previous studies (Bernet et al., 2014; Brack et al., 2007; Chakkalakal, Jones, Basson, & Brack, 2012; Conboy, Conboy, Smythe, & Rando, 2003; Cosgrove et al., 2014; Lee et al., 2013; Shavlakadze, McGeachie, & Grounds, 2010; Sousa‐Victor et al., 2014), we found that muscle regeneration after cardiotoxin (CTX) injury is delayed in male aged animals (Figure 1a–c), as shown by the distribution of the cross‐sectional area (CSA; Supporting information Figure S1A–D), the mean CSA (Figure 1b), the increase in necrotic fiber content at Day 8 post‐CTX (Figure 1c), and the muscle mass alterations during the regeneration process (Figure 1d).

To test, whether innate immune responses, in addition to previously identified age‐related changes in satellite cell function, could also contribute to impairment in muscle regeneration, we set out to characterize the cellular dynamics of the myeloid cell infiltration in uninjured tissues and during muscle regeneration. We could detect increased expression level of macrophage activation markers in aged uninjured and regenerating muscles compared to young controls (Figure 1f). Next, we isolated myeloid cells from CTX‐injured TA muscles at Days 0, 1, 2, 4, and 6 after the injury. In an interesting manner, we found a statistically significant increase in the number of invading myeloid cells (CD45^+^) in the aged versus young muscles at Day 4 (repair phase; Figure 1e). These findings suggested the existence of age‐related changes in the cellular composition and differentiation profile of the infiltrating myeloid cells. Indeed, the ratio of Ly6C^high^ F4/80^low^ (inflammatory) macrophages to Ly6C^low^ F4/80^high^ (repair) macrophages in injured muscle between young versus aged animals showed remarkable differences (Figure 1g–h), suggesting a delay in the phenotypic transition of infiltrating myeloid cells to repair macrophages in the aged muscles.

**Figure 2:**
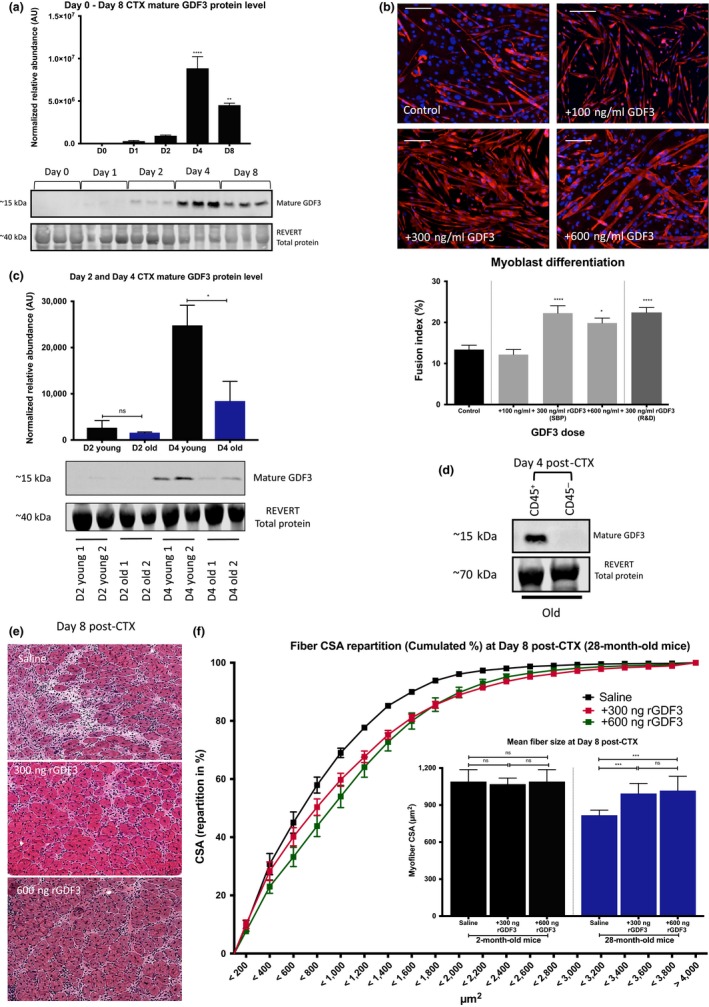
GDF3 induces myoblast differentiation and improves the kinetics of regeneration in aged animals. (a) GDF3 protein expression in whole‐muscle lysates of regenerating TA muscles from young adult male mice (2‐month‐old) at indicated time points (D, day) post‐CTX injury. Three representative biological replicates are shown for each time point (lower panel), and mean normalized signal values to total protein of each group are shown (upper panel) (*n* = 6). REVERT total protein was used for loading control and signal normalization. (b) Immunofluorescence against desmin (red) and DAPI (blue) shows a drastic enhancement of myotube formation in the presence of recombinant (r) GDF3 in primary myoblasts (*n* = 4). Representative images from 100, 300, and 600 ng/ml rGDF3‐treated myoblasts are shown (upper panel). Lower panel shows the fusion index of primary myoblasts in the presence of various concentrations of recombinant GDF3 (*n* = 4). Two different sources of the protein were used. SBP stands for the in‐house Sanford Burnham Prebys Protein Core produced version and R&D for the commercially available one. (c) Decreased GDF3 protein expression in whole‐muscle lysates of regenerating muscles from aged (28‐month‐old) mice at different time points (D, day). Two representative biological replicates are shown for each group (lower panel) with mean normalized signal values to total protein of each group shown in the upper panel (*n* = 6). REVERT total protein was used for loading control and signal normalization. (d) GDF3 protein expression in CD45^+^ and CD45^‐^ cells isolated at Day 4 post‐CTX injury from aged (23‐month‐old) mice. REVERT total protein was used for loading control. (e, f) Improvement in regeneration by administration of recombinant GDF3 (300 and 600 ng) in young (2‐month‐old) or aged (28‐month‐old) male animals (*n* = 6 per group). (e) H&E‐stained images from aged treated mice, and (f) cumulated and mean CSA (right panel) measurements are shown. In all bar graphs, bars represent mean ± *SEM*

Several members of the TGFβ family (Egerman et al., 2015) are known regulators of muscle regeneration, whose members are secreted by repair macrophages acting in a paracrine manner (Massague, Cheifetz, Endo, & Nadal‐Ginard, 1986; McPherron, Lawler, & Lee, 1997), including GDF3 (Varga et al., 2016). We selected GDF3 for a *proof‐of‐concept* experiment to evaluate whether the observed impaired phenotypic transition in macrophage phenotype from inflammatory to repair type (Patsalos et al., 2017) can contribute to age‐related delay in muscle regeneration. In line with previous findings (Varga et al., 2016), GDF3 protein expression in whole‐muscle lysates of CTX‐injured young mice showed a pronounced induction, which peaked at Day 4 (Figure 2a), at the time when inflammation subsides, and regenerative processes start to dominate within the injured muscle. In controlled in vitro conditions, addition of recombinant GDF3 (using either an in‐house recombinant protein or a commercially available one) in primary myoblasts induced a robust effect in myotube formation (Figure 2b upper panel) and a pronounced increase in their fusion index (Figure 2b lower panel). These results confirmed the positive impact of GDF3 on the muscle regeneration process. In an important way, and in line with the delayed macrophage phenotype transition (Figure 1g–h), we found decreased GDF3 protein levels at Day 4 post‐CTX in the aged mice compared to young controls validating our initial hypothesis (Figure 2c). In an important way, GDF3 expression was detectable only in the CD45‐positive (hematopoietic) compartment of aged muscles (Figure 2d), suggesting that the repair macrophages from aged mice are the predominant source of GDF‐3.

To determine whether introducing recombinant GDF3 back into the aged animals can restore regeneration, we used a single intramuscular dose of 300 ng rGDF3 at Day 4 post‐CTX. To a remarkable degree, this treatment restores the morphological features of the aged muscle at Day 8 (Figure 2e,f) while treating young mice with rGDF3 had no obvious enhancing effect (myofiber size increase or faster regeneration; Figure 2f). These findings suggest that the endogenous physiological levels of GDF3 are sufficient for proper regeneration, and regeneration must be impaired in order for the rGDF3 treatment to have an effect. Taken together, these results highlight that GDF3 alone could compensate for the age‐related decrease in repair macrophage and improve the kinetics of muscle repair.

In conclusion, our findings suggest that aging has a negative effect on the ability of macrophages to perform their phenotypic transition, leading to reduced production of growth factors (including GDF3), and this impacts the muscle regeneration potential. This work provides strong evidence that the immune axis should be considered for future therapeutic interventions.

## CONFLICT OF INTEREST

None declared.

## AUTHOR CONTRIBUTIONS

A.P. and L.N. conceptualized the study. A.P., T.H., M.P., Z.S., and L.N involved in methodology. A.P., Z.S., and T.H. validated the data. A.P., T.H., Z.S., M.P., and L.N. analyzed the data. A.R, P.C, and L.N. provided resources. A.P., M.H., I. R., Z.S., and L.N. wrote and drafted the original manuscript. A.P., Z.S., and L.N. wrote, reviewed, and edited the manuscript. A.P. visualized the data. P.C. and L.N. acquired funding information. L.N. supervised the study.
