# Supplementary figures and images for: In vivo GDF3 administration abrogates aging related muscle regeneration delay following acute sterile injury
Source: Aging Cell. 2018 Jul 12;17(5):e12815. doi: 10.1111/acel.12815 (PMC6156497; doi:10.1111/acel.12815)

Supplementary Figure 1.

A

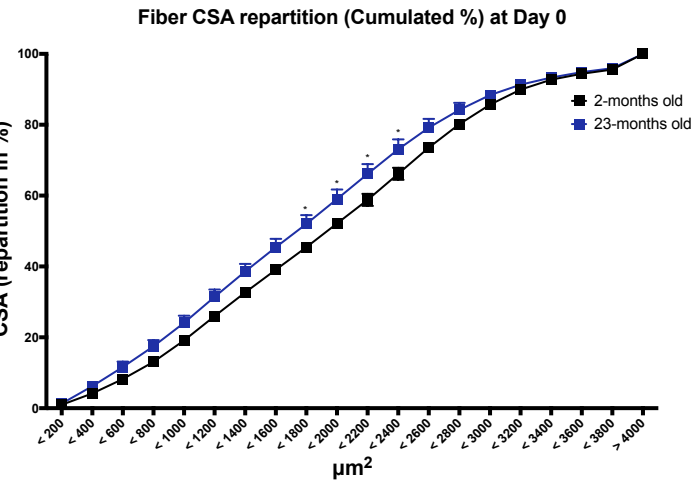

B

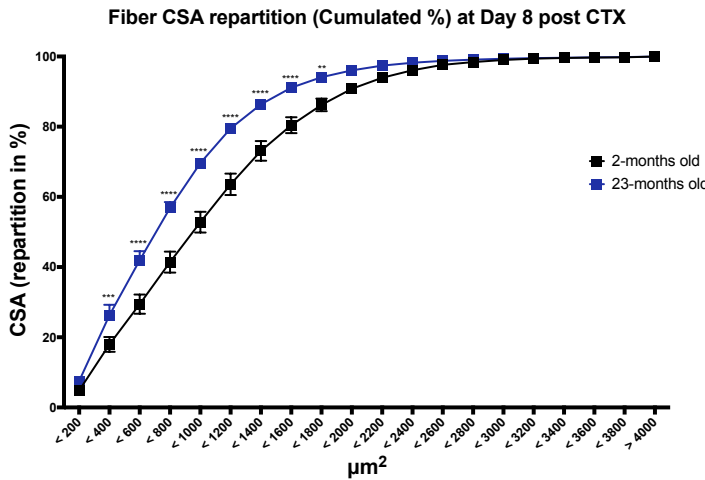

C

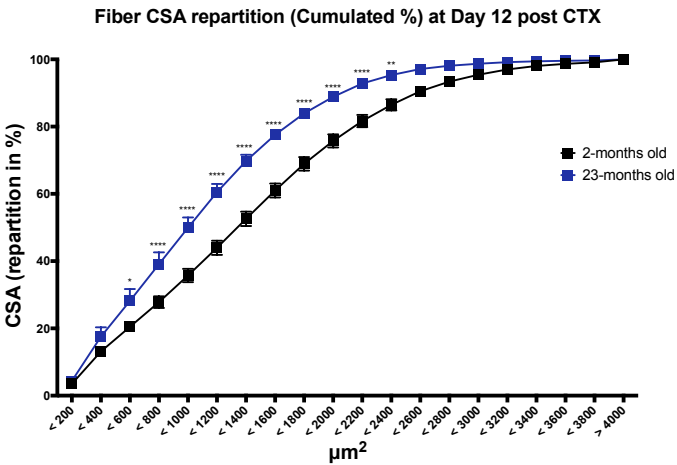

D

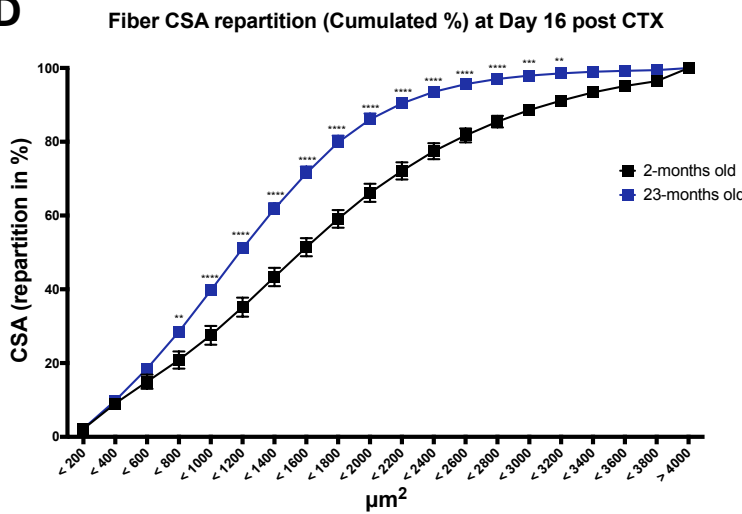

Supplement: Supplementary file 1 [file ACEL-17-e12815-s001.pdf]
