# Supplementary material for: In vivo GDF3 administration abrogates aging related muscle regeneration delay following acute sterile injury
Source: Aging Cell. 2018 Jul 12;17(5):e12815. doi: 10.1111/acel.12815 (PMC6156497; doi:10.1111/acel.12815)
Supplement: Supplementary file 2 [file ACEL-17-e12815-s002.docx]

**Materials and Methods**

**Ethical approval:**

All animal experiments were carried out in accordance with guidelines prescribed by the Institutional Animal Care and Use Committee (IACUC) at Sanford Burnham Prebys Medical Discovery Institute.

**Mice:**

Wild type C57BL/6J male control mice were obtained from the Jackson Laboratories and bred under specific-pathogen free (SPF) conditions. Experiments were conducted on young adult (2-3-month-old) and old (23-28-month-old) male mice obtained by GlaxoSmithKline. All mice were fed rodent chow *ad libitum*. When necessary and for tissue collection mice were euthanized by either isoflurane overdose (adjusted flow rate or concentration to 5% or greater) or CO_2_exposure (adjusted flow rate 3 L/min) in accordance to the Sanford Burnham Prebys Medical Discovery Institute’s IACUC guidelines. When indicated recombinant GDF3 was administrated intramuscularly on a single dose at day 4 post CTX under anesthesia.

**Muscle injury and rGDF3 administration:**

Mice were anaesthetized with isoflurane (adjusted flow rate or concentration to 1,5%) and 50 µl of cardiotoxin (12x10^-6^ M in PBS) (from Latoxan) was injected in the *tibialis anterior* (TA) muscle. Muscles were recovered for flow cytometry analysis at day 1 to day 6 post-injury or for muscle histology at day 8 post-injury. In some experiments, 300ng or 600ng recombinant (r) GDF3 in 50 ul saline, was injected intramuscularly at day 4 post-CTX into each TA muscle. For the production of the in-house GDF3 protein, the mature peptide of GDF3 was cloned into a pET20b(+) plasmid and produced in E.Coli. Recombinant mature GDF3 was His-tag purified, underwent endotoxin removal, lyophilized, and then freeze dried.

**Histological analysis of muscle regeneration:**

Muscles were removed and snap frozen in nitrogen-chilled isopentane (–160°C). 8 µm thick cryosections were cut and stained with hematoxylin-eosin (H&E). For each histological analysis, at least 5 slides (per condition) were selected where the total regenerative region within the CTX injured TA muscle was at least 70%. For each TA, myofibers in the entire injured area were counted and measured. H&E muscle sections were scanned with Mirax digital slide scanner and the cross-sectional Area (CSA) was measured with HALO software (Indica Labs). CSAs for these samples are reported in µm^2^. Quantitative analysis of necrotic/phagocytic was performed using the Panoramic Viewer software and was expressed as a percentage of the total number of myofibers. Necrotic/phagocyted myofibers were defined as pink pale patchy fibers that are invaded by basophil single cells (macrophages).

**Isolation of macrophages from muscle:**

Fascia of the TA was removed (this excludes most of the tissue resident macrophages from our analysis). Muscles were dissociated in RPMI containing 0.2% collagenase B (Roche Diagnostics GmbH) at 37°C for 1 hour and filtered through a 100 µm and a 40 µm filter. CD45^+^ cells were isolated using magnetic sorting (Miltenyi Biotec). For FACS, macrophages were treated with Fcγ receptor blocking antibodies and with 10% normal rat serum: normal mouse serum 1:1 mix, then stained with a combination of PE-conjugated anti-Ly6C antibody (HK1.4, eBioscience), APC-conjugated F4/80 antibody (BM8, eBioscience) and FITC-conjugated Ly6G antibody (1A8, Biolegend). Ly6C^high^ F4/80l^ow^ macrophages, Ly6C^low^ F4/80^high^ macrophages and Ly6G^high^ Ly6C^med^ F4/80^-^ neutrophils were quantified. In each experiment, compared samples were processed in parallel to minimize experimental variation. Cells were analyzed on a MoFlo Astrios EQ cell sorter (Beckman Coulter) and data analysis was performed using FlowJo V10 software.

**Western Blot:**

GDF3 protein expression was measured using western blot analysis. Homogenates were prepared from CTX injected TA muscles using a TissueLyser II (Qiagen) and stainless-steel beads in Tissue Extraction Reagent 1 (Thermo Fisher Scientific) with a protease inhibitor cocktail (Thermo Fisher Scientific). GDF3 was targeted using a rabbit monoclonal anti-GDF3 primary antibody (ab108617, Abcam) at 1:1000 dilution in Odyssey Blocking Buffer with 0.1% Tween20 overnight at 4°C. Total protein was measured using REVERT total protein stain (LI-COR). Bands were visualized using an Odyssey Digital Infrared Imaging System (LI-COR) and quantified using Odyssey Application Software version 3.0 (LI-COR).

**Primary myoblast cell culture:**

Primary murine myoblasts were obtained from TA muscle and cultured using standard conditions in DMEM/F12 (Gibco Life Technologies) containing 20% FBS and 2% Ultroser G (Pall, Inc). Briefly, TA muscles of young mice were opened and cleared of nerves/blood vessels/fascia etc. Muscle preparations were lightly digested with collagenase and the resulting cells were plated then serially expanded. For differentiation studies, MPCs were seeded at 30000 cell/cm^2^ and incubated for 3 days with conditioned medium containing 2% horse serum or with 2% horse serum medium containing recombinant GDF3 (in house by SBP Protein Core or R&D 958-G3-010). Cells were then incubated with anti-desmin antibodies (32362 Abcam), in combination with a Cy3-conjugated secondary antibody (Jackson Immunoresearch Inc).

**Image Capture and Analysis for Myoblast Cultures:**

Fusion index (for myogenic cells) was calculated as the number of nuclei within myotubes divided by the total number of nuclei. Image analysis and nuclei number was estimated using the ImageJ software.

**RNA isolation:**

Total RNA was isolated from whole TA muscles with TRIZOL reagent according to the manufacturer’s recommendation. 20ug glycogen (Ambion) was added as carrier for RNA precipitation.

**RT-qPCR:**

Transcript quantification was performed by quantitative real-time RT (reverse transcriptase) PCR (polymerase chain reaction) using SYBR Green assays. RT-qPCR results were analyzed with the standard delta Ct method and results were normalized to the expression of RPLP0. Heatmap was generated based on log_10_-transformed FC values with R software package pheatmap.

Primer sequences used in transcript quantification are as follows:

**Table S1.** Primers for qRT-PCR detection of mRNA (SYBR GREEN)

| **Gene** | **Forward primer** | **Reverse primer** |
| --- | --- | --- |
| **Rplp0 (36b4)** | agggcgacctggaagtcc | cccacaatgaagcattttgga |
| **Fbxo32** | agtgaggaccggctactgtg | gatcaaacgcttgcgaatct |
| **Myod1** | agcactacagtggcgactca | ggccgctgtaatccatca |
| **Igfbp4** | gacacctcgggaggaacc | aagaggtcttcgtgggtacg |
| **Trim63** | tgacatctacaagcaggagtgc | tcgtcttcgtgttccttgc |
| **Lrp4** | cttggtcagccatgtgtcc | ccagtccgtccagtagatcc |
| **Myh1** | tctgcagacggagtcaggt | ttgagtgaatgcctgtttgc |
| **Myh4** | tggccgagcaagagctac | ttgatgaggctggtgttctg |
| **Atf4** | tcctgaacagcgaagtgttg | acccatgaggtttcaagtgc |
| **Cath** | gtggactgttctcacgctcaag | tccgtccttcgcttcatagg |
| **Ampd3** | gcggagaaggtgtttgcta | cagtcttgttgtgttggcatc |
| **Adgre1 (F4/80)** | ggaggacttctccaagcctatt | aggcctctcagacttctgctt |
| **Ccl2** | catccacgtgttggctca | gatcatcttgctggtgaatgagt |
| **Cd36** | TAGTAGAACCGGGCCACGTA | GCAGAATCAAGGGAGAGCAC |
| **Cd68** | gacctacatcagagcccgagt | cgccatgaatgtccactg |
| **Cd80** | tgctgctgattcgtctttca | gattcggtcttcagactcatcttc |
| **Cd86** | GGCTTGGCAATCCTTATCTTT | GGCAGATATGCAGTCCCATT |
| **Cxcl10** | gctgccgtcattttctgc | tctcactggcccgtcatc |
| **Il1b** | agttgacggaccccaaaag | agctggatgctctcatcagg |
| **Mrc1** | ccacagcattgaggagtttg | acagctcatcatttggctca |
| **Tgfb1** | tggagcaacatgtggaactc | gtcagcagccggttacca |

**Statistics:**

All animal experiments were performed using at least four biological replicates and repeated at least 3 times. For the myoblast fusion assay at least 1000 cells were counted for each experiment. Student’s t-tests and 2-way ANOVA analyses were performed in GraphPad Prism 7 and P < 0.05 was considered significant (P<0.05=*, P<0.01=**, P<0.001=***, P<0.0001=****). Mean and SEM values are shown in graphs.
